# Incidence and Prognostic Factors for Colectomy in Acute Severe Ulcerative Colitis with Concomitant CMV Infection

**DOI:** 10.3390/diseases13100321

**Published:** 2025-10-01

**Authors:** Konstantina Kitsou, Konstantina Chalakatevaki, Georgios Kokkotis, Evgenia Papathanasiou, Vassiliki Kitsou, Spyridon Michopoulos, Evanthia Zampeli, Giorgos Bamias

**Affiliations:** 1 3rd Academic Department of Internal Medicine, GI Unit, University of Athens, Sotiria Hospital, Attica, 11527 Athens, Greece; konkitsou@med.uoa.gr (K.K.); gkokkot@gmail.com (G.K.); vassosgkp@gmail.com (V.K.); 2 National and Kapodistrian University of Athens, Attica, 11527 Athens, Greece; 3 Gastroenterology Department, Alexandra General Hospital, Attica, 11528 Athens, Greece; evgeniapapathanasiou@gmail.com (E.P.); michosp5@gmail.com (S.M.); evazamb@gmail.com (E.Z.)

**Keywords:** ulcerative colitis, ASUC, CMV, colectomy, prognostic factor

## Abstract

Background/Objectives: Cytomegalovirus (CMV) is an opportunistic pathogen, complicating acute severe ulcerative colitis (ASUC), and its role in ASUC prognosis remains a debate. This study aims to report the rates and identify predictors for colectomy at 12 months, following an episode of ASUC with concomitant CMV colonic infection. Methods: This is a retrospective cohort study of patients with ASUC and CMV colonic infection confirmed by PCR or Immunohistochemistry. Baseline clinical, biochemical, endoscopic and disease-related characteristics were recorded. Patients were followed-up for 12 months to calculate the one-year colectomy rate. Predictors of colectomy were identified via multivariate logistic regression. Results: Forty-five cases of CMV colonic infection in 37 patients with ASUC were recorded [66.7% men, mean age: 47.0 years (SD = 18.5)]. At diagnosis, 20% were on monotherapy with advanced treatment and 37.8% on advanced treatment plus corticosteroids and/or immunomodulators. Twenty-three (51.1%) were receiving corticosteroids, while 17.8% did not receive any immunosuppressive agent. Forty (88.9%) patients were treated with ganciclovir and valganciclovir and one (2.2%) with foscarnet for at least 21 days. Eleven patients (24.4%) required colectomy, two (4.4%) during their initial hospitalization and nine (20%) during the follow-up period. The recurrence of CMV was recorded in nine (20.9%) cases, three of which required colectomy. Patients with hemoglobin < 12 g/dL (*p* = 0.023) and patients on vedolizumab at diagnosis (*p* = 0.050) had a higher probability of colectomy. Conclusions: We report a 25% one-year colectomy rate in our cohort with ASUC and superimposed CMV colonic infection. At baseline, anemia and vedolizumab treatment were associated with a higher probability of colectomy.

## 1. Introduction

Ulcerative colitis (UC) is a major form of Inflammatory Bowel Disease (IBD) that causes chronic intestinal inflammation, which is restricted to the colon. Although substantial advances in the treatment options for patients have occurred in recent years, the natural history of UC can still be complicated by colonic inflammation of extreme severity, a condition that is referred to as acute severe ulcerative colitis (ASUC) [1]. This represents a medical emergency requiring hospitalization as it is characterized by increased risk for adverse outcomes, including colectomy and increased mortality [2]. It has been estimated that, among patients with UC, up to 25% will present with ASUC during the course of their disease [3].

Cytomegalovirus (CMV), a large double-stranded DNA virus, is a member of the Herpesviridae family, with a high estimated seroprevalence between 39 and 96% in various populations [4]. After primary infection, CMV employs multiple immune evasion mechanisms, allowing it to persist in life-long latency. However, primary infection and CMV reactivations are typically asymptomatic in the immunocompetent host [5]. In contrast, in the immunocompromised host, the reactivation of CMV may cause end-organ disease, including CMV colitis [6].

CMV frequently arises as an opportunistic pathogen in patients with UC. On the one hand, colonic inflammation is associated with increased mucosal permeability, thus rendering the colonic mucosa vulnerable to the translocation of microorganisms. On the other hand, treatment with broad and targeted immunotherapies affects mucosal immunity, further adding to the suppression of mucosal defense mechanisms [7]. Regarding ASUC, CMV reactivation has been estimated to occur in between 21% and 34% of patients and correlated to steroid resistance [8]. Risk factors for CMV reactivation in UC include patients’ demographic features as well as the administered regimes [8].

The significance of the effect of concomitant CMV infection in patients with ASUC on disease outcomes, including the risk of colectomy, remains a subject of debate among specialists. Accumulating evidence indicates an aggravating role in the course of the disease and its prognosis [9]. In fact, concomitant CMV colonic infection and ASUC have been correlated to unfavorable outcomes, such as prolonged hospitalization, rescue treatment administration and increased risk of re-admission during follow-up [10]. Whether CMV concomitant infection or reactivation exerts similar effects on the risk of colectomy and whether the detection of CMV serves as a surrogate marker of disease severity or can be postulated as an innocent bystander regarding these outcomes remain controversial at the moment [10,11]. Whilst the aggravating effect of CMV infection on ASUC prognosis regarding adverse outcomes other than colectomy has been verified with no clear correlation to the risk of colectomy, other groups have demonstrated an increased risk in these patients, with ganciclovir offering limited advantages in the long-term prevention of colectomy [11]. Antiviral administration in patients with steroid-refractory UC leads to lower colectomy risk, underscoring the potential impact of CMV colonic infection in colectomy-free survival [12]. According to the European Crohn’s and Colitis Organization, patients with moderate-to-severe UC flares should be screened for concomitant CMV infection and receive prompt antiviral treatment [13].

In this retrospective descriptive study, we aimed to identify the baseline characteristics of patients with ASUC and concomitant CMV colonic infection and report on their management and outcomes in two Tertiary Centers. Furthermore, we aimed to elucidate the clinical significance of CMV colonic infection in ASUC patients in terms of the rates of colectomy during the first year following diagnosis and detect prognostic factors for this outcome.

## 2. Materials and Methods

Study design: This is a descriptive retrospective cohort study. The study population consisted of adult patients with confirmed UC, followed-up at the Gastroenterology Units of two tertiary General Hospitals, “Sotiria Hospital” and “Alexandra General Hospital” in Athens, Greece. We included patients hospitalized between 1 January 2017 and 12 December 2023, whose data stemming from the medical records of the hospitals were anonymously entered in the IBD patient registry of the two centers. The study entry point was defined as the day of diagnosis of CMV infection, and each patient was followed-up for a 12-month period.

Patients with ASUC underwent a colonoscopy to evaluate endoscopic severity according to Trulove and Witt’s criteria [14]. Biopsies were obtained to rule out colonic CMV infection in those with refractory disease to immunosuppressive agents [15] and with severe mucosal inflammation, such as deep ulcers.

Immunohistochemistry (IHC) and/or qualitative Polymerase Chain Reaction (PCR) and pathology examination were performed on the obtained tissues. Since qualitative PCR may detect both latent and active CMV particles, a quantitative assay has gained popularity in the establishment of CMV diagnosis [7,16,17]. Patients with confirmed colonic CMV infection [positive PCR or IHC or typical “owl eye appearance” inclusion bodies in colonic mucosal biopsies stained with hematoxylin and eosin] constitute this cohort.

We recorded each patient’s gender, age, disease duration, disease extension according to the Montreal classification (E1: ulcerative proctitis; E2: left sided [distal] colitis; E3: extensive colitis) [18] and the presence of extraintestinal manifestations. Clinical activity was assessed with the non-invasive, patient-reported outcomes (PROs) of the MAYO score (“Bowel movement” and “Rectal bleeding”) [19] and Simple Clinical Colitis Activity Index (“Urgency”) [20], and endoscopic activity was assessed with the Mayo endoscopic score and the Ulcerative Colitis Endoscopic Index of Severity (UCEIS) [21]. Prior and concomitant treatments for UC were categorized as non-immunosuppressive (mesalazine) and immunosuppressive (corticosteroids, azathioprine, methotrexate, biologics and small molecules). The baseline and sequential values of hemoglobulin, platelets, white blood cells, CRP and albumin were also recorded. Finally, we recorded the type of antiviral treatment that was administered for the management of CMV infection.

Study endpoints: The primary endpoint was colectomy during the follow-up period, while the secondary endpoint was to describe potential prognostic factors of this outcome.

Statistical analysis: Normally distributed variables are presented with their mean values and their standard deviation, while non-normally distributed variables are presented with their median value and their interquartile range. Univariate Cox regression models were used to identify factors related to the occurrence of colectomy. The factors identified from the univariate models (with a cut-off *p*-value = 0.1) were included in a multivariate Cox regression model. Fischer’s exact test was used for the comparison of proportions. A *p*-value of 0.05 was set as the limit of statistical significance. All statistical analyses were performed with the statistical package SPSS v23 (IBM, Armonk, NY, USA).

## 3. Results

### 3.1. Study Population

Forty-five (n = 45) cases of colonic infection with CMV were reported in 37 patients with ASUC. Six patients experienced more than one recurrence of CMV infection. In particular, four patients had one recurrence (in total two episodes of CMV/ASUC), and two had three separate episodes. None of the patients was pregnant at the time of CMV diagnosis, and no gestation occurred during the follow-up period.

Most cases were observed in men (66.7%) with a mean age of 47.0 years (SD 18.5) and median UC duration of 3 years (IQR 1–7.5) (Table 1). Regarding disease extent, 31 (68.9%) cases were extensive, and 14 (31.1%) were left-sided colitis (Table 1). The diagnosis of CMV colonic infection was established through PCR in colonic tissue obtained via endoscopic biopsies in 21 (46.7%) cases, IHC in 24 (53.3%), and by the detection of affluent “owl eye appearance” inclusion bodies in 3 (6.7%).

Regarding treatment at the time of diagnosis of CMV infection, nine (20.0%) patients were on monotherapy with advanced treatment [five with anti-Tumor Necrosis Factor (anti-TNF) (11.1%), three with vedolizumab (6.7%) and one with tofacitinib (2.2%)], one with azathioprine and eight (17.8%) with corticosteroids. Eleven (24.4%) were on a combination of corticosteroids plus advanced treatment [four with anti-TNF (8.9%), four with vedolizumab (8.9%) and three with tofacitinib (6.7%)], three (6.7%) were on a combination of infliximab with azathioprine and four (8.9%) were on a combination of infliximab, immunomodulator and corticosteroids. Regarding patients’ history of advanced treatment refractory disease, all 7 patients were biologic-naïve when they received vedolizumab, while 8/19 of patients on anti-TNF or tofacitinib and 8/38 of non-vedo patients had a history of biologic refractory disease. Eight patients (17.8%) were not on immunosuppressive therapy.

Treatment for CMV infection was administered in 41 (91.1%) patients, who received antiviral agents for at least 21 days. Twenty-six (57.8%) were treated with ganciclovir for 14 days during their hospitalization and commenced at-home valganciclovir for 7 days, and fourteen (31.1%) were treated with ganciclovir for 21 days. Foscarnet administration was required in one case (2.2%), due to CMV resistance to ganciclovir. No statistically significant difference was observed in the colectomy rates between the patients who received antiviral treatment (11/41) and those who did not (0/4), Fischer’s exact test *p* = 0.559. Accordingly, no statistically significant difference was observed in the colectomy rates between the patients that were treated with ganciclovir and valganciclovir (7/26) and those who were treated with ganciclovir for 21 days (4/14), Fischer’s exact test *p* = 0.999.

### 3.2. Colectomy Rates During the Follow-Up Period

The colectomy rate during the first year following concomitant CMV colonic infection and ASUC was 24.4% (n = 11). Two patients (4.4%) required colectomy during their initial hospitalization and nine (20%) during the one-year follow-up period due to disease that was refractory to maintenance treatment.

In 43 cases in which immediate colectomy was not required, 18 (41.9%) received maintenance treatment with an anti-TNF agent, 6 (14.0%) with ustekinumab, 4 (9.3%) with vedolizumab, 3 (7.0%) with tofacitinib and 1 (2.3%) with risankizumab.

During the follow-up period, the recurrence of CMV infection was observed in 10 cases (23.3%), and 3 patients underwent colectomy. In particular, recurrence occurred in 5/18 (27.8%) of patients treated with anti-TNF [2 of them were on concomitant azathioprine], 2/4 (50%) with vedolizumab, 1/1 (100%) with tofacitinib and 2/5 (40%) with mesalamine.

### 3.3. Prognostic Factors of Colectomy Following CMV Intestinal Infection

In univariate Cox regression models, age greater than 54 years (*p* = 0.026), hemoglobin less than 12 g/dL (*p* = 0.062), serum albumin less than 3.8 g/dl (*p* = 0.072), PRO-rectal bleeding = 3 (*p* = 0.087), PRO-bowel movement = 3 (*p* = 0.021), vedolizumab as treatment at diagnosis (*p* = 0.034) and the presence of deep ulcers at the endoscopy (*p* = 0.084) were associated with a higher probability of colectomy in the first year (Table 2).

In the multivariate Cox regression model, statistical significance for the prediction of colectomy was maintained for hemoglobin less than 12 g/dL (OR = 8.73; 95% CI: 1.22–62.48; *p* = 0.031) and vedolizumab as treatment at diagnosis (OR = 24.46; 95% CI: 2.31–259.50; *p* = 0.008) (Table 2).

**Figure 1:**
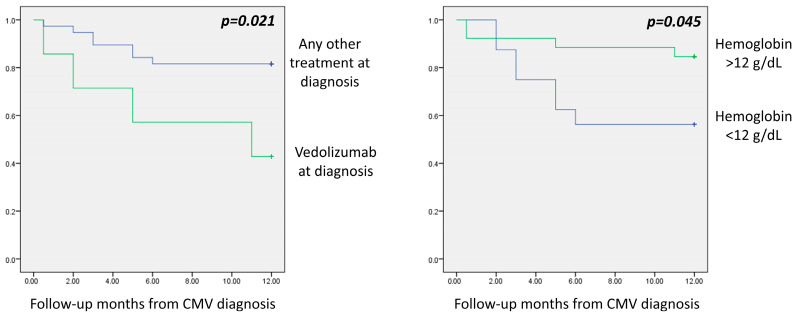
Vedolizumab treatment at diagnosis and hemoglobin levels less than 12 g/dL were independently associated with colectomy during the first year of follow-up (Kaplan–Meier Log Rank: *p* = 0.021 and *p* = 0.045, respectively).

Colectomy was needed in 57.1% (4/7) of patients treated with vedolizumab at diagnosis in comparison to 22.6% (7/31) of those treated with something other than vedolizumab. Furthermore, it was needed in 43.8% (7/16) of patients with hemoglobin less than 12 g/dL in comparison to 15.4% (4/26) of those with hemoglobin greater than 12 g/dL (Figure 1).

No statistically significant difference was observed in the colectomy rates between the patients who were diagnosed through PCR (3/18) and those who were diagnosed with IHC (8/24), Fischer’s exact test *p* = 0.299. No statistically significant difference was observed in the colectomy rates between the patients who received antiviral treatment (11/41) and those who did not (0/4), Fischer’s exact test *p* = 0.559. Accordingly, no statistically significant difference was observed in the colectomy rates between the patients that were treated with ganciclovir and valganciclovir (7/26) and those who were treated with ganciclovir for 21 days (4/14), Fischer’s exact test *p* = 0.999.

## 4. Discussion

In the present study, we report a 25% colectomy rate during one year of follow-up in patients who were admitted for a diagnosis of acute severe ulcerative colitis and found to have concomitant colonic infection with CMV. Furthermore, we identified low hemoglobin and baseline treatment with vedolizumab as independent risk factors for colectomy in this patient population.

The colectomy rate of 25% that was observed in our cohort is lower than those reported in previous cohorts of patients with ASUC and concomitant CMV. Colectomy rates of 33.3% [22] and 35.5% [23] were shown in two other reports. The differences between our findings and those of other studies may be attributable to various factors. First, the aforementioned studies included a small number of patients. Second, the follow-up periods were substantially longer, up to 5.5 years, which may have allowed for more colectomies to be performed [22,23]. Finally, our report includes patients in the era of biologics, which means that more therapeutic options are available, as compared to earlier works. Notably, most available studies were conducted in the pre-biologic era, a period characterized by limited therapeutic options for both the acute management of ASUC and the maintenance of remission following the initial episode of hospitalization. This is further supported by the fact that the colectomy rates in our cohort are closer to those observed overall in patients with ASUC [24,25,26,27], irrespectively of the presence of infection with CMV, as such studies also refer to the pre-biological time.

The extent to which direct colon damage caused by the virus is superimposed on the baseline inflammation of UC or whether CMV detection merely serves as a surrogate marker of severe disease in patients with ulcerative colitis remains difficult to distinguish and continues to be an open question in the literature. The observation that most colectomies were performed long after the index admission supports the latter explanation. All patients in our cohort who required colectomy had received antiviral therapy, and CMV was expected to have been cleared, consistent with the findings in patients who underwent repeat endoscopies prior to surgery. At present, concomitant CMV infection in patients with a UC flare should be regarded as a poor prognostic factor for colectomy, even though the exact mechanism remains unclear.

In our cohort of patients, we identified factors that were associated with an increased probability of adverse outcomes, in particular colectomy. The first factor that was independently predictive of colectomy during one year of follow-up was treatment with vedolizumab at the diagnosis of CMV infection. Currently, data on the potential correlation between vedolizumab and CMV colonic infection and its outcomes are limited [28,29]. Vedolizumab selectively blocks α4β7 integrin on immune cells and prevents adhesion to its cognate molecule Mucosal Addressin Cell Adhesion Molecule-1 (MAdCAM-1) on the endothelial cells of the gastrointestinal tract, thus interfering with inflammatory cell trafficking to the inflamed gut [30].

Multiple immune cell subsets demonstrate reduced α4β7 integrin expression under treatment with vedolizumab, including circulating B-cells, Natural Killer (NK) cells, dendritic cells and various CD4+ T-cell subsets, such as Th2- and Th17-cells, as well as effector and memory T-cells [31,32]. Vedolizumab-treated patients have lower counts of colonic naïve B- and T-cells and dendritic cells [33]. These data offer a biologically plausible mechanism for the increased severity and unfavorable outcomes of CMV colonic infection, in the presence of α4β7 integrin blockade. In particular, antiviral defense against colonic CMV is characterized by the accumulation of antigen-specific effector memory T-cells in the colonic mucosa and Th17 and Th2 immune responses locally [34,35], whilst dendritic cells are key components of antiviral defense against CMV [36]. Consequently, it is reasonable to postulate that compromised antiviral pathways may potentially lead to more severe colitis in patients with ASUC/CMV colitis. However, since only a small fraction of our patients in this cohort received vedolizumab at the time of the CMV diagnosis, the verification of this finding is required in larger scale studies. This will also aid in the clarification of the extent of the influence of this agent in the prognosis of ASUC complicated with CMV colonic infection, while mechanistic studies would be important in the elucidation of the immunological background behind its effects. It should be noted, however, that the majority of patients that developed concomitant ASUC and CMV infection received immunosuppressive treatment at the time of diagnosis, which may also affect the severity of colitis. Corticosteroids and thiopurines have been identified as additional risk factors of CMV reactivation in patients with IBD and have been found to upregulate CMV replication, leading to higher rates of CMV reactivation in patients with IBD [34]. Indeed, almost half of the patients in our cohort were under corticosteroid treatment at diagnosis.

Anemia was identified as an additional independent risk factor for colectomy in the first year after CMV infection. It has long been a well-established risk factor of poor prognosis in patients with ASUC, in general, and is also included as a parameter of Truelove and Witts’ criteria for ASUC [14,37,38,39]. Therefore, patients with anemia could be considered for a more aggressive approach in their treatment, such as intensified induction. In a previous study, Hirayama Y et al. reported that extensive colitis was an independent risk factor for CMV infection in patients with ulcerative colitis [40]. Similarly, extensive colitis was present in most of our patients with combined ASUC and CMV infection, indicating a positive feedback loop between mucosal inflammation and infection with CMV. On the one hand, the extensive mucosal inflammation characteristic of extensive colitis may contribute to higher intestinal viral burden via the loss of local defense mechanisms, including increased intestinal permeability and mucosal vulnerability to pathogens and antigenic triggers. On the other hand, CMV infection directly interferes with TNF-a production, a main mediator of UC, which may amplify inflammatory pathways at the colon [7,40].

Our study has certain limitations. First, the descriptive and retrospective nature of our study and the lack of a control group of patients with ASUC without concomitant CMV infection are important limitations in the recognition of significant associations and allow only for the description of our cohort. However, we were able to identify independent risk factors of colectomy in the setting of ASUC with CMV infection when we compared the characteristics of patients with ASUC that required colectomy to those that did not, and thus, we believe that the significance of our findings overcomes this shortcoming. Additionally, the retrospective nature of our cohort led to heterogeneity among the treatment regimens used for the treatment of CMV infection, and the small sample size in our cohort did not allow us to investigate stricter endpoints than colectomy, such as sustained clinical remission. Both these shortcomings could be overcome in future prospective studies with a larger population. Finally, our definition of CMV colitis was not uniform but consisted of histological, immunohistochemical or molecular criteria. In fact, half of our CMV diagnoses were established through qualitative PCR, which may potentially overestimate CMV positivity, instead of IHC, which could explain the difference from previous colectomy rate estimations made by other groups [23,41].

## 5. Conclusions

Patients with ASUC and CMV primary infection or reactivation, especially those with anemia and vedolizumab baseline treatment, are at increased colectomy risk. CMV infection should be promptly identified during ASUC workup as it reflects a significant predictor of adverse outcomes. Further studies should focus on differential gene expression analysis comparing ASUC patients with and without concomitant CMV infection, which may help elucidate distinct inflammatory pathways associated with CMV, potentially validating its contribution to a more severe disease trajectory and offering definitive therapeutic algorithms.

## Figures and Tables

**Table 1:** Demographics and disease-related characteristics of 45 CMV infections.

Age in Years [Mean (SD)]	47.0 (18.5)
Male [n (%)]	30 (66.7)
Disease duration [median (IQR)]	3 (1–7.5)
Montreal [n (%)]	
E2	14 (31.1)
E3	31 (68.9)
EIMs [n (%)]	12 (26.7)
Family history [n (%)]	2 (4.4)
Treatment at infection [n (%)]	
Corticosteroids	23 (51.1)
Immmunomodulators	8 (17.8)
Biologics	26 (57.8)
Anti-TNF	15 (33.3)
Vedolizumab	7 (15.6)
Tofacitinib	4 (8.9)
Clinical scores [median (IQR)]	
PRO-Rectal bleeding	2 (2–3)
PRO-Bowel movement	2 (2–3)
Urgency	1 (1–1.8)
Endoscopic findings	
Endoscopic MAYO [median (IQR)]	3 (2–3)
UCEIS [median (IQR)]	6 (5–6)
Deep ulcers [n (%)]	25 (55.6)
Laboratory findings	
Hemoglobin g/dL [mean (SD)]	12.5 (1.9)
Platelets × 10^9^/L [mean (SD)]	327 (100)
White blood cells × 10^9^/L [mean (SD)]	9748 (3267)
CRP mg/L [median (IQR)]	15.8 (5.9–36.1)
Albumin g/dL [mean (SD)]	3.8 (0.6)

Immunomodulators: azathioprine, methotrexate.

**Table 2:** Cox regression of predictors for colectomy during severe ulcerative colitis with CMV infection.

	Univariate			Multivariate		
	OR	95% CI	*p*	OR	95% CI	*p*
Age less than 54 years	4.05	1.18–13.85	0.026	0.69	0.14–3.48	0.648
Receiving vedolizumab at diagnosis	3.80	1.11–12.99	0.034	**24.46**	**2.31–259.5**	**0.008**
PRO-Rectal Bleeding = 3	3.19	0.85–12.04	0.087	1.72	0.21–14.07	0.613
PRO-Bowel movement = 3	6.12	1.32–28.39	0.021	5.42	0.42–69.67	0.195
Deep ulcers at endoscopy	3.86	0.84–17.89	0.084	2.65	0.48–14.75	0.267
Hemoglobin < 12 g/dL	3.23	0.94–11.08	0.062	**8.73**	**1.22–62.48**	**0.031**
Albumin < 3.8 g/dL	3.47	0.90–13.44	0.072	2.40	0.44–12.98	0.310

The results that remained statistically significant after the implementation of a multivariate model are shown in bold.

## Data Availability

The raw data supporting the conclusions of this article will be made available by the authors on request.

## Abbreviations

UC	Ulcerative Colitis
IBD	Inflammatory Bowel Disease
ASUC	Acute Severe Ulcerative Colitis
CMV	Cytomegalovirus
IHC	Immunohistochemistry
PCR	Polymerase Chain Reaction
PROs	Patient-Reported Outcomes
UCEIS	Ulcerative Colitis Endoscopic Index of Severity
Anti-TNF	Anti-Tumor Necrosis Factor
MAdCAM-1	Mucosal Addressin Cell Adhesion Molecule-1
NK cells	Natural Killer Cells
